# Meta analysis of clinical efficacy of acupoint application in the treatment of irritable bowel syndrome

**DOI:** 10.4314/ahs.v24i4.44

**Published:** 2024-12

**Authors:** Qingbo Wang, Lili Zhao, Junhong Liu, Li Chen, Baoxia Zhang, Qiong Zhang, Yike Lu, Yang Gao, Xue Zheng, Zongqing He, Shuangshuang Jing

**Affiliations:** 1 The first affiliated Hospital of Henan University of traditional Chinese Medicine, Zhengzhou, Henan, 450000; 2 School of Acupuncture and Massage, Henan University of traditional Chinese Medicine, Zhengzhou, Henan, 450018

**Keywords:** Acupoint application, irritable bowel syndrome, diarrhea type, constipation type, mixed-type

## Abstract

**Purpose:**

The clinical efficacy of acupoint application in the treatment of irritable bowel syndrome (IBS) was evaluated by Meta-analysis.

**Method:**

Computer searched Chinese and English databases for the randomized controlled trials (RCTs) and quasi-randomized controlled trials (CCTs) of TCM external therapy, acupoint application, TCM external application, navel sticking and their combination therapy in the treatment of IBS. The search period is from the establishment of the database to December 2022. The literature was screened independently by 2 researchers according to the standard of nano-ranking, and the data of the other 5 researchers were proofread, screened and extracted. After that, the bias risk of the included study was evaluated, and the data were analysed by RevMan 5.4. software.

**Result:**

(1) A total of 1842 patients, were included in 25 randomized controlled trials, including the acupoint application treatment group (n=928) and conventional therapy control group (n = 914).

(2) The quality of the literature method shows that there are 5 high-quality literatures with a score of 4-7, 20 low-quality literatures with a score of 1-3 and few high-quality literatures;

(3) In terms of effectiveness, compared with the western medicine control group, the total odds ratio OR [95 % CI] of the total effective rate of the acupoint application treatment group was 4.77 [3.68, 6.20], and the difference was statistically significant (*P* < 0.05). Shenque, Zhongwan, Pishu and Zusanli are the most commonly used.

(4) In terms of literature bias, 2 studies used envelopes to hide, which belonged to “low risk”; 9 studies were blindly implemented and evaluated as “unclear”; and 6 studies were rated as “high risk” because cases fell off but were not reported. The funnel plot shows that the study is scatter symmetrical, the probability of publication bias is small, and the conclusion is reliable.

**Conclusion:**

Acupoint application can improve the effective clinical rate of IBS with fewer adverse reactions, better patient compliance and fewer adverse reactions, but it still needs to be confirmed by high-quality multicenter, large sample randomized controlled trials.

## Introduction

Irritable bowel syndrome (IIBS) is a common gastrointestinal functional disease characterized by diarrhea or constipation, abdominal pain and abdominal distension.

It is a combination of chronic abdominal pain associated with a change in the frequency or form of stool. Its persistent or intermittent episodes lack an available organic disease explanation 1.

The overall incidence of IBS is increasing 2.

The prevalence of IBS in China ranges from 0.82% to 5.67%, the onset is related to the living environment, psychological factors, dietary structure, etc. The onset of the population is mainly young and middle-aged people, more women than men and there is a tendency for family gathering 3. Modern medicine relies primarily to symptomatic treatment for antidiarrheal, pain relief and improvement of gastrointestinal function, supplemented by other drug treatments for adjusting gastrointestinal flora and regulating emotions; Although it can improve the symptoms, it lacks satisfactory long-term effect 4.

At present, western medicine treatment is mainly symptomatic treatment, including antispasmodic, analgesic, antidiarrheal, promoting intestinal dynamics, laxative, regulation of intestinal flora, antidepressant, psycho-behavioral therapy, etc., but there are disadvantages such as more difficult to cure, high recurrence rate, and large adverse reactions 5.

In recent years, clinical studies of traditional Chinese medicine have found that acupuncture point sticker has a definite clinical effect in the treatment of irritable bowel syndrome, and this disease belongs to one of the dominant diseases of traditional Chinese medicine 6.

In recent years, the results of Meta-analysis have proved that the clinical efficacy of traditional Chinese medicine such as acupuncture and traditional Chinese medicine in treating IBS is better than that of western medicine and the safety is better. 7

However, there are many kinds of traditional Chinese medicine (TCM) treatments and there are few in-depth studies on the clinical efficacy of some external TCM treatments; Acupoint application, as one of the traditional external treatment methods of traditional Chinese medicine, applies drugs to the corresponding acupoints to regulate the yin and yang of human viscera and prevent the occurrence and transmission of diseases by stimulating acupoints and drug absorption. 8

Therefore, this study searched the literatures of clinical randomized controlled trials (RCTs) and semi-randomized controlled trials (CCTs) of acupoint application in the treatment of irritable bowel syndrome in Chinese and English databases and analysed their clinical efficacy by Meta analysis, so as to provide a reference for the clinical treatment of IBS in the future. The summary is as follows.

## Materials and methods

### Retrieval strategy

Computer retrieval databases include China National Knowledge Infrastructure (CNKI), VIP Information (VIP), WanFang Data, China Biomedical Documentation Service (CBM); English databases PubMed, Embase, Ovid, Cochrane Library. Search for RCT and CCT literatures on acupoint application therapy for IBS published from the establishment of the database to December 2022. Chinese search terms include: sticking, acupuncture point sticker, acupuncture point sticker, dog-fu sticking, irritable bowel syndrome, external treatment of traditional Chinese medicine, summer treatment for winter diseases, diarrhea type, constipation type, navel sticking, navel sticking, traditional Chinese medicine sticking, Navel application, Shenque point application, randomized controlled trials, quasi-randomized controlled trials, etc.; English search terms include: Irritable bowel syndrome, randomized controlled trail, stick moxibustion, quasi-randomized controlled trails, moxibustion, acupuncture, etc.

### Inclusion and exclusion criteria

**Research type:** Acupoint application was used to treat RCT and CCT of IBS.

**Research objects:** The patients belong to the diagnostic criteria of IBS, and the clinical types are diarrhea type, constipation type, mixed type and unclassified type 9.

**Intervention measures:** The treatment group was treated with different acupuncture point sticker therapy, such as moxibustion, umbilical application, powder, external application of ointment, etc.; the control group was mainly treated with western medicine and integrated traditional Chinese and western medicine.

**Outcome indicators:** Clinical effective rate of acupoint application in the treatment of IBS.

**Inclusion criteria:** a. The study type was a randomized controlled trial, and the language was Chinese or English; b. The study subjects were clearly diagnosed with IBS-D, and age, gender, and TCM evidence type were not required; c. The intervention in the observation group was the use of one of the six acupuncture therapies mentioned above, and the intervention in the control group was another acupuncture therapy or western medicine; e. The outcome index was the total clinical efficiency.

**Exclusion criteria:** a. Only the treatment group, but no control group.; b. Treatment group without acupoint application therapy; c. Animal Experiment, cell Experiment and mechanism Study; d. Theoretical research such as literature review; e. Repeated publications; f. Non-IBS research literature.

### Literature screening and data extraction

The literature retrieval was completed by two researchers (He Zongqing and Jing Shuang Shuang). Search terms include: acupuncture, electric-acupuncture, acupuncture therapy, moxibustion, sham acupuncture, catgut implantation at acupoint, needle warming, irritable bowel syndromes, IBS, IBS-D, randomized controlled trial and RCT etc. According to the proposed inclusion and exclusion criteria, download and screen documents that may meet the criteria by browsing the title and abstract of the literature. If there are different opinions, the third researcher (Zheng Xue) will make a ruling, and two researchers (Wang Qingbo and Chen Li) will independently extract data from the final included literature and input them into the Revman 5.4.1 software.

### Risk of bias assessment of included studies

According to the bias risk assessment tool recommended by the Cochrane manual, two researchers (Zhang Qiong and Zhang Baoxia) evaluated it.

### Statistical processing

The total clinical effective rate belongs to two-category variable data, taking the ratio (Odds Ratio, OR) as the effect and calculating the 95% confidence interval of OR (95% Confidence interval, CI). Firstly, quality assessment and quality assessment visualization were carried out, and the literature data and risk bias assessment data were imported into RevMan 5.4.1 software, and then analysed and visualized. Then, the heterogeneity test between the results of each study was carried out, and the test level was 0.05. If there was statistical homogeneity between the study results (*P* > 0.1, *I*^2^ < 50%), the analysis method used a fixed effect model. On the contrary, the statistical heterogeneity among the results of each study (P ≤ 0.1. *I*^2^ ≥ 50%) was analysed by random effect model.

According to the causes of heterogeneity, subgroup or sensitivity analyses were performed if necessary. The potential publication bias was analysed by funnel chart. In terms of combined effects, Continuous data use mean and standard deviation (SD) as measurement indicators, including mean deviation (MD), standardized mean deviation (SMD) and weighted mean deviation (WMD), For dichotomous data, relative risk (RR) or odds ratio (OR) were used as measures. The above results all give 95% confidence interval (Confidence Interval, CI). If the heterogeneity is too large, Meta-analysis cannot be carried out. Only descriptive analysis can be carried out. Sensitivity analysis can be used to evaluate the reliability and stability of meta-analysis results. To show the contents of statistical analysis, a forest Plot can be used, and when the number of Meta analysis studies ≥ 10:00, funnel Plot) is used to observe whether the results are biased.

## Result

### Results of literature screening

**Figure 1:**
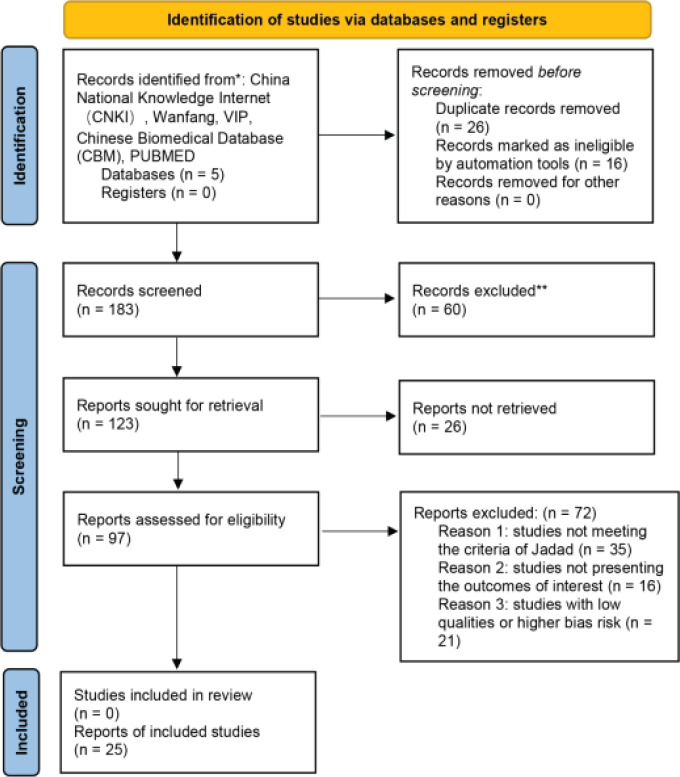
Search strategy and result

Input keywords and subject words were searched in various databases commonly used at home and abroad, and a total of 182 Chinese literatures and 1 English literature were obtained. A total of 60 duplicate literatures (including 1 English literature with the same content as the published Chinese literature) were screened out by the Note Express 3.3 software, and the remaining 123 literatures were included. By reading the title and abstract of the literature, and using the modified Jadad scoring scale to classify, including 5 high-quality literatures with 4-7 points, 92 low-quality literatures with 1-3 points, and 72 literatures that do not meet the requirements or have a high risk of bias. 25 literatures that finally meet the requirements will be re-read and screened. Finally, the data of the 25 documents were extracted 6-30 and included in the statistical analysis of RevMan 5.4.1 software (Figure 1).

### Include the basic characteristics of the literature

All the 25 RCT literatures included were carried out in China and published in Chinese journals. A total of 1842 patients, including 928 in the treatment group and 914 in the control group, with a minimum sample size of 21 and a maximum sample size of 73. The shortest course of treatment is 10 days, and the longest course is 60 days. Fifteen studies used acupuncture point sticker combined with other therapies to compare Western medicine for IBS, 25 reported the total clinical response rate and 16 reported symptom scores.

### Meta-analysis results

#### Quality evaluation and publication bias of included studies

In terms of random sequence generation, the random allocation scheme of 6 studies was unclear and evaluated as “unclear”; 25 studies mentioned random words, and all of them used random number table method, all of which were evaluated as “low risk”. In terms of allocation scheme hiding, two studies used envelopes to hide, which were evaluated as “low risk”. Concealment of allocation scheme was unclear in 6 studies and was rated as “unclear”. In terms of blind implementation, 17 studies mentioned single-blind or double-blind and were evaluated as “low risk”. In 9 studies, blind implementation was unclear and evaluated as “unclear”. In the analysis of results, none of the 7 studies mentioned the issue of blinding by analysts, and they were rated as “unclear”. In terms of outcome data integrity, 6 studies showed case shedding but were not reported, which were evaluated as “high risk”, 14 studies did not mention case shedding and were evaluated as “low risk”, and the remaining 25 studies were evaluated as “low risk” without incomplete data. In terms of selective reporting of outcome indicators, the selective reporting of 5 studies was unclear and was rated as “unclear”. For other biases, 10 studies were unclear and were rated as “unclear”. There were 20 studies with a total Jadad score ≤ 3 and 5 studies with a total score of ≥ 4. See Table 1.

**Table 1:** Basic characteristics of included literature

Name and year of included study authors	sample size Treatment group / Control group	Diagnostic criteria	Intervention measures		Course of treatment	Jadad score	Outcome index

			Treatment group	Control group			
Yang Zhongting 2020^[10]^	73/73	(1); (2)	Invigorating spleen and warming kidney cataplasm paste navel	Placebo Pabu patch umbilical cord	4 weeks	6.0	1; 3; 4; 5
Ji Chunyan 2021^[11]^	50/50	(2); (3)	Piveronium bromide tablets + Bifidotetralian viable bacteria + Meridian Liuzhu Nazhifalei fire moxibustion + Acupoint application	Piveronium bromide tablets + Bifidotetralian viable bacteria + Thunder fire moxibustion + Acupoint application	4 weeks	4.0	1; 2; 3; 4;
Sun Jianyu 2020^[12]^	25/25/25	(2); (3)	Oral administration of Yigan Jianpi recipe + Acupoint application	Oral administration of Yigan Jianpi recipe	4 weeks	4.0	1; 2; 3; 6; 7
Zhou Tao 2020^[13]^	73/73	(1); (2)	Invigorating spleen and warming kidney poultice	Placebo spleen and kidney poultice	4 weeks	4.0	1; 2; 3; 4; 5
Li Shujin 2020^[14]^	36/36	(2); (3)	Acupoint application	Oral administration of Enterobacteriaceae Thai recipe	4 weeks	3.0	1; 4; 6
Li Jianwen 2020^[15]^	30/30/30	(3); (4)	Acupuncture combined with acupuncture point sticker	Pinaverium Bromide Tablets	10 days	3.0	1
Jin Yueqin 2017^[16]^	28/27	(1); (4)	acupuncture + Acupoint application	Trimebutine Maleate Capsules	4 weeks	4.0	1; 3
Li Xinzhi 2021^[17]^	30/31	(2); (3)	Acupoint application 9-11AM	Acupoint application except 9-11AM time period	4 weeks	3.0	1; 2;
He Wanting 2019^[18]^	25/25	(2); (3)	Lactobacillus acidophilus tablets + Acupoint application	Lactobacillus acidophilus tablets	4 weeks	3.0	1; 4;
Luan Yanhe 2019^[19]^	30/30	(2); (3)	Shenling Baizhu powder addition and subtraction + Acupoint application	Shenling Baizhu powder addition and subtraction + acupuncture	4 weeks	3.0	1; 2; 3
Jin Yueping 2021^[20]^	30/30	(3); (4)	Flavored kudzu root soup + Live Bacillus licheniformis capsule + Trimex	Flavored kudzu root soup + Live Bacillus licheniformis capsule + Trimex	2 weeks	3.0	1; 2; 3;
Zhan Daowei 2016^[21]^	28/27	(1); (4)	Acupuncture + Acupoint application	Trimex maleate pudding capsules	4 weeks	3.0	1; 3
Wang Menglin 2019^[22]^	30/30	(2); (3); (4)	Warm soup to replenish the void + Acupoint application	Pivitropium bromide tablets	4 weeks	3.0	1; 2
Wang Yuanyuan 2020^[23]^	31/30/30/30	(2); (3)	Acupoint application + Hot buns	Ginseng spleen tablets	2 weeks	3.0	1; 2; 4; 5
Chen Defeng 2020^[24]^	35/35	(1); (2)	Acupoint application	bolus for regulating middle energizer + pill of four miraculous drugs + montmorillonite powder	30 days	3.0	1; 2; 4
Xiao Beiyan 2019^[25]^	30/30	(2); (3)	Acupoint application + Acupoint Application of	Pivitropium bromide tablets + Loperamide tablets	4 weeks	3.0	1; 2; 3
Liang Biqi 2013^[26]^	30/30	(1); (4)	pain and diarrhea	Trimebutine maleate capsules	10 days	3.0	1
Hua hanbing2020 ^[27]^	30/30/30	(2); (3)	Maziren pill decoction + Acupoint application	Maziren pill decoction	2 weeks	3.0	1
Han Songhua 2018^[28]^	60/60	(2)	Lei's Qingli accumulation method + Acupoint application	Otiamium bromide tablets + clostridium butyricum tablets	4 weeks	3.0	1; 2; 3
Li Lin 2012^[29]^	28/28	(1); (4)	Spleen and liver patch acupuncture point patch + Addition and subtraction of pain and diarrhea	Addition and subtraction of pain and diarrhea	4 weeks	3.0	1; 2; 4
Wang Shihong 2010^[30]^	58/41	(1); (4)	Turbidity + Acupoint application	Solid bowel antidiarrhea pills	60 days	3.0	1
Shi Hao 2021^[31]^	50/50/50	(1); (2)	Milli-fire needles + Acupoint application	Pivitropium bromide tablets	4 weeks	3.0	1; 2; 3;
Song Xiong 2021^[32]^	60/60	(2); (3)	Trimebutine maleate capsules + Flavored Shisei granules + Acupoint application	Trimebutine maleate capsules	8 weeks	3.0	1; 2; 4;
Liu Binghan 2018^[33]^	23/23	(1); (4)	acupuncture + Acupoint application	Trimebutine Maleate Tablets	4 weeks	3.0	1; 2; 3; 7
Xu Qing 2014^[34]^	30/30	(1); (4)	Ginseng medicinal soup + Acupoint application	Tonic pills	30 days	3.0	1; 2

Note: (1)Total clinical effective rate (2) Chinese medicine syndrome scores (3) IBS Quality of Life Scale (IBS-QOL (4) IBS symptom severity scale (IBS-SSS (5) Stool character questionnaire IBS-DSQ (6) Chinese Irritable Bowel Syndrome Patient Reported Outcome Scale PRO (SSDPRO) (7) Recurrence rate (1) IBS Rome III (2) Consensus of TCM experts in diagnosis and treatment of irritable bowel syndrome (3) IBS Rome IV (4) Guiding principles for Clinical Research of New drugs of traditional Chinese Medicine (trial).

#### Meta analysis results of clinical total effective rate

**Figure 2:**
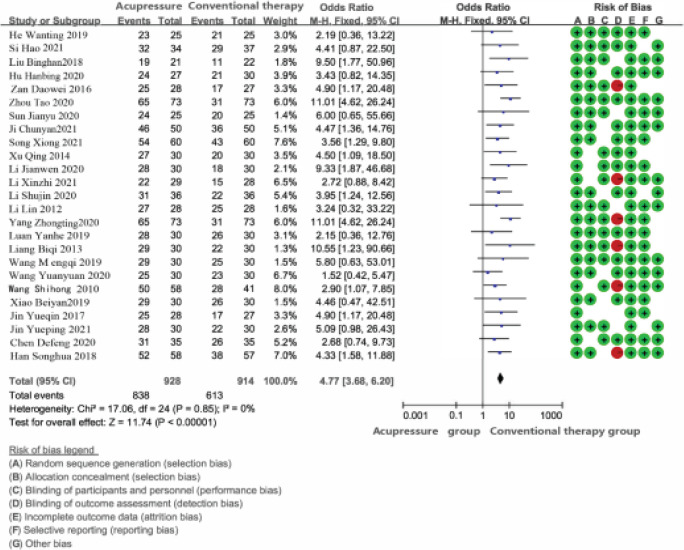
The clinical effective rate of acupomt application in the treatment of IBS forest map and bias risk analysis map

A total of 25 studies reported the total clinical effective rate, of which 15 studies evaluated the total effective rate according to the guiding principles of Clinical Research on New drugs of traditional Chinese medicine. 3 studies used “consensus on Integrated traditional Chinese and Western Medicine diagnosis and treatment of irritable bowel syndrome (2011)” to evaluate the total effective rate and 4 studies used “consensus on traditional Chinese Medicine diagnosis and treatment of irritable bowel syndrome (2010)” to evaluate the total effective rate. Three studies evaluated the total effective rate according to the standard of clinical disease diagnosis based on cure and improvement. After the heterogeneity test, there was no heterogeneity among the studies (*I*^2^ = 0 %, *P* = 0.85), and the study type was RCT study, and the data type was binary variable, so the fixed effect model and OR combined effect size was used. Meta-analysis results show that: The difference between the control group and the experimental group was statistically significant [OR = 4.77, 95% CI (3.68, 2.60), *P* < .00001]. It can be seen that acupoint application or traditional Chinese medicine combined with acupoint application can improve the total clinical effective rate of IBS more than western medicine. See Figure 2.

### Safety evaluation

Twelve studies (12) observed the adverse reactions of acupoint application in the treatment of IBS, one of which showed obvious adverse reactions. The common adverse reactions in the control group were thirst, constipation, drowsiness and so on. The common adverse reactions in the treatment group were local skin flushing, itching and other discomforts, and the symptoms disappeared after withdrawal.

In other studies, no adverse reactions were reported in the acupoint application treatment group and the western medicine control group.

### Bias analysis

**Figure 3:**
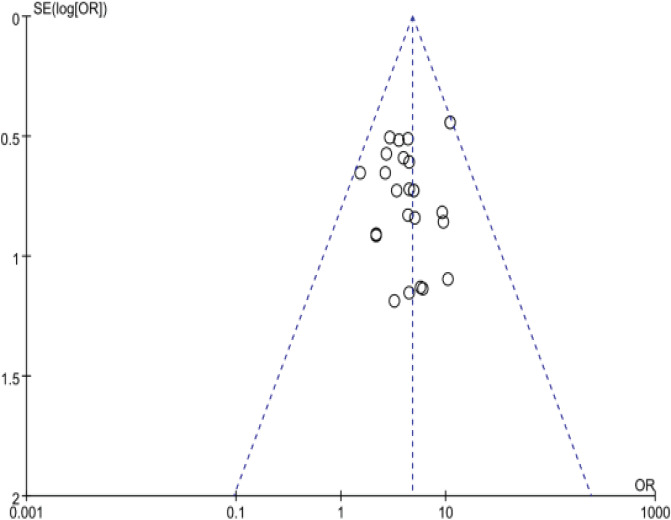
Clinical effective rate of acupoint application in the treatment of IBS results funnel chart

The clinical effective rate of acupoint application in the treatment of IBS is an inverted funnel. The figure is basically an inverted funnel, and the scattered points are basically symmetrical, suggesting that the probability of publication bias is small and the conclusion is reliable, as shown in figure 3.

## Discussion

Irritable Bowel Syndrome (IBS) is a functional gastrointestinal disease divided into diarrhea-type irritable bowel syndrome, constipated irritable bowel syndrome, mixed irritable bowel syndrome and amorphous irritable bowel syndrome according to clinical features 35. Modern medicine believes that the pathogenesis of IBS is closely related to visceral hypersensitivity, abnormal gastrointestinal motility, intestinal flora disorder, abnormal brain-gut-axis regulation, gastrointestinal infection and other factors 36-37. The incidence of IBS is similar to the “diarrhea”, “constipation”, “abdominal pain” and other diseases of traditional Chinese medicine, and its causes are mainly deficiency of the body, external evil, internal injury diet (Poor diet, unclean food and drink, and a partial diet), and internal emotional injury, and its symptoms involve spleen and stomach deficiency, heart and spleen deficiency, wet and evil spleen, liver and spleen irregularities, liver qi and spleen, intestinal damp heat, spleen and kidney yang deficiency, etc^38^. Acupoint application therapy is a unique external therapy of traditional Chinese medicine developed through the dual effects of acupoints and drugs under the guidance of the meridian theory of traditional Chinese medicine and on the basis of syndrome differentiation and treatment. It is loved by clinicians and patients with the characteristics of being cheap, convenient, safe and effective, which not only avoids the first-pass effect of the digestive tract, but also reduces the stimulation of drugs to the digestive tract 8. Patients can better accept, and compliance is better than western medicine therapy. In this study, we collected the relevant literature in the past 30 years and included the literature strictly according to the standard of nano-platoon. The results of Meta-analysis are reliable and can be used as a reference for clinical application.

In this paper, it is found that the safety and total clinical efficiency of acupuncture point patch treatment IBS are higher than those of Western medicine therapy (*P* < 0.05), and the most commonly used acupuncture points include: Shen Que, Zhong, Spleen Yu, Zu Sanli and other acupuncture points, which may provide a reference for the clinical treatment of IBS. Studies have shown that acupoint application or acupoint application combined with traditional Chinese medicine therapy does have adverse reactions in the treatment of IBS, but its occurrence can be reduced or eliminated by drug withdrawal or active treatment. Due to the insufficient amount of data in this study, the sample content is not high, and the evaluation indicators are few, the effectiveness and safety of acupuncture points on IBS need to be studied in depth. This study confirmed the effectiveness of acupoint application on IBS from the perspective of evidence-based medicine, and proved that as an appropriate TCM technology for treating IBS, it is worth popularizing and applying.

**The advantages of this study are as follows:**

1). Only the clinical efficacy of acupoint application in the treatment of IBS was studied, without paying attention to other outcome indicators such as symptom scores, stool traits, and life function scores, which reduced the risk of bias in the results.
2). In the meta-analysis, the related therapies such as acupuncture or TCM combined acupuncture point patches were compared with clinical first-line treatment methods such as Western medicine, and the results showed that TCM combined acupuncture point patches or simple acupuncture point patches were superior to Western medicine therapy.
3). In this study, TCM-related therapy is subdivided into fire needle, heat-sensitive moxibustion, traditional Chinese medicine powder and acupoint application, in order to analyse better the possible factors that the effective clinical rate of TCM combined therapy is better than that of western medicine therapy.

In this study, the Meta-analysis of the clinical effective rate of acupoint application in the treatment of IBS and the funnel plot of the clinical effective rate and publication bias between the two groups were better, but the results still had the following deficiencies:

1). Clinical research is not standardized, especially pay less attention to sample size estimation, blind method, follow-up, loss of follow-up and so on.
2). Diversification of interventions: Different studies have different acupoints and medication, there is no uniform standard, needs further exploration
3). The efficacy assessment is more subjective and the adverse reactions are less recorded.
4). There are many missing hidden methods in the implementation of blind method, and there are very few literatures about blind method and correct application of hidden method.
5) There are many problems in random grouping method, such as not using systematic random grouping method and so on. Therefore, the conclusions of this study still need to be confirmed by more randomized controlled trials with reasonable design, strict execution, multicenter and large samples.

## Conclusions

In this study, TCM-related therapy is subdivided into fire needle, heat-sensitive moxibustion, traditional Chinese medicine powder and acupoint application, in order to analyse better the possible factors that the effective clinical rate of TCM combined therapy is better than that of western medicine therapy. Acupoint application can improve the effective clinical rate of IBS with fewer adverse reactions, better patient compliance and fewer adverse reactions.
